# Omega-3 Fatty Acids and Depression: Scientific Evidence and Biological Mechanisms

**DOI:** 10.1155/2014/313570

**Published:** 2014-03-18

**Authors:** Giuseppe Grosso, Fabio Galvano, Stefano Marventano, Michele Malaguarnera, Claudio Bucolo, Filippo Drago, Filippo Caraci

**Affiliations:** ^1^Department of Clinical and Molecular Biomedicine, Section of Pharmacology and Biochemistry, University of Catania, Viale A. Doria 6, 95125 Catania, Italy; ^2^Department of “G.F. Ingrassia”, Section of Hygiene and Public Health, University of Catania, Via S. Sofia 85, 95123 Catania, Italy; ^3^Department of Educational Sciences, University of Catania, Via Teatro Greco 84, 95124 Catania, Italy; ^4^IRCCS Associazione Oasi Maria S.S.-Institute for Research on Mental Retardation and Brain Aging, Via Conte Ruggiero 73, Enna, 94018 Troina, Italy

## Abstract

The changing of omega-6/omega-3 polyunsaturated fatty acids (PUFA) in the food supply of Western societies occurred over the last 150 years is thought to promote the pathogenesis of many inflammatory-related diseases, including depressive disorders. Several epidemiological studies reported a significant inverse correlation between intake of oily fish and depression or bipolar disorders. Studies conducted specifically on the association between omega-3 intake and depression reported contrasting results, suggesting that the preventive role of omega-3 PUFA may depend also on other factors, such as overall diet quality and the social environment. Accordingly, tertiary prevention with omega-3 PUFA supplement in depressed patients has reached greater effectiveness during the last recent years, although definitive statements on their use in depression therapy cannot be yet freely asserted. Among the biological properties of omega-3 PUFA, their anti-inflammatory effects and their important role on the structural changing of the brain should be taken into account to better understand the possible pathway through which they can be effective both in preventing or treating depression. However, the problem of how to correct the inadequate supply of omega-3 PUFA in the Westernized countries' diet is a priority in order to set food and health policies and also dietary recommendations for individuals and population groups.

## 1. Introduction 

**Figure 1:**
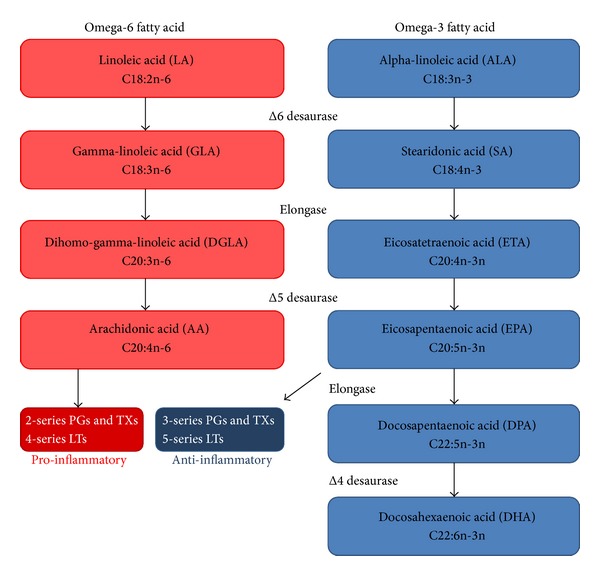
Biosynthesis of the principal polyunsaturated fatty acids and their metabolism.

Polyunsaturated fatty acids (PUFA) are fatty acids that contain more two or more carbon-carbon double bonds not saturated with hydrogen atoms at multiple (poly) locations within the molecule. PUFA can be classified into various groups by their chemical structure in omega-3 and omega-6 fatty acids: the omega-3 PUFA (also called *ω*-3 fatty acids or n-3 fatty acids) refers to a group of PUFA in which the first double bond is 3 carbons from the end (omega) carbon atom of the molecule; the omega-6 (also referred to as *ω*-6 fatty acids or n-6 fatty acids) are a family of PUFA that have in common a final carbon–carbon double bond in the n-6 position, that is, the sixth bond, counting from the methyl end [1]. Omega-3 PUFA are synthetized by dietary shorter-chained omega-3 fatty acid alpha-linolenic acid (ALA) to form the more important long-chain omega-3 fatty acids: eicosapentaenoic acid (EPA) and docosahexaenoic acid (DHA) (Figure 1). Omega-6 PUFA derive from linoleic acid (LA), which can be converted also into the 18-carbon gamma linolenic acid (GLA), and the 20 carbon arachidonic (AA) and dihomo-gamma-linolenic acids (DGLA) (Figure 1). Both LA and ALA are considered essential fatty acids because mammalian cells are unable to synthesize these fatty acids from simpler precursors. Omega-3 PUFA have been long investigated for their anti-inflammatory effects in inflammatory-related diseases [2]. Omega-6 PUFA can be converted into AA and then metabolized into the omega-6 eicosanoids, which has proinflammatory action (Figure 1). On the other hand, omega-3 PUFA increase EPA in the cell membrane. This competes with AA for enzymatic conversion into its own metabolites, the omega-3 derived eicosanoids. These are less active and can partly oppose or antagonize the proinflammatory actions of the omega-6 eicosanoids. Noninflammatory eicosanoid balance is maintained throughout the body by way of a homeostatic balance between omega-3 and omega-6 fatty acids in cell membranes. Eicosanoid balance then exerts a “downstream” balancing influence on cytokines.

In the context of the modern human lifestyle and diet, an absolute change of omega-6/omega-3 in the food supply of Western societies has occurred over the last 150 years [4]. Although the eicosanoid metabolites of EPA are crucial to provide anti-inflammatory effects by balancing the potentially proinflammatory eicosanoid metabolites of the omega-6 AA, a ratio of omega-6/omega-3 of 15 : 1 to 16.7 : 1, instead of 1 : 1 as is the case with animal and prehistoric human being has been reported [5]. Thus, the existing balance between omega-6 and omega-3 PUFA for the years during the long evolutionary history of the human being has rapidly changed over a short period of time, not accompanied by corresponding genetic changes. In other words, humans living in modern societies are exposed to a nutritional environment that differs from their genetic constitution. Not by chance, omega-3 PUFA have been considered of particular interest for the treatment of certain forms of chronic diseases [6]. In particular, many epidemiological and experimental studies emphasized their possible role in the prevention or treatment of depressive disorders. Due to evidence from animal and human studies reporting that omega-3 deficiency leads to impaired neuronal function (especially of serotoninergic and dopaminergic neurotransmitters) and altered inflammatory status, the biological plausibility of the effects of the omega-3 PUFA raised several hypotheses although merely speculative [7]. The aim of this study was to review the current knowledge about the association between the omega-3 PUFA and depression, taking into account both the epidemiological and experimental studies. The biological mechanisms of action of omega-3 PUFA in preventing or treating depression have been also reviewed.

## 2. Epidemiological Aspects Regarding Depressive Disorders and Diet

### 2.1. Burden of the Disease

Depression is a mental disorder characterized by sadness, loss of interest in activities, and decreased energy. Other symptoms include loss of confidence and self-esteem, inappropriate guilt, thoughts of death and suicide, diminished concentration, and disturbance of sleep and appetite. There are multiple variations of depression that a person can suffer from: (i) depressive episode involves symptoms such as depressed mood, loss of interest and enjoyment, and increased fatigability, categorized as mild, moderate, or severe; (ii) bipolar affective disorders typically consist of both manic and depressive episodes separated by periods of normal mood. Diagnostic criteria for a major depressive episode (DSM-IV) include a depressed mood, a marked reduction of interest or pleasure in virtually all activities, or both, lasting for at least 2 weeks. In addition, 3 or more of the following must be present: gain or loss of weight, increased or decreased sleep, increased or decreased level of psychomotor activity, fatigue, feelings of guilt or worthlessness, diminished ability to concentrate, and recurring thoughts of death or suicide. Particularly when long-lasting and with moderate or severe intensity, depression may become a serious health condition. In about 20% of cases, however, depression follows a chronic course with low rates of remission, especially when adequate treatment is not available. The recurrence rate for those who recover from the first episode is around 35% within 2 years and about 60% at 12 years. The recurrence rate is higher in those who are more than 45 years of age. Depression is associated with significant disability [8] and with excess mortality, particularly increasing the risk of cardiovascular diseases [9]. By 2020, depression is projected to be the second leading cause of disease burden worldwide after heart disease. Depression is associated with dysregulation of circadian rhythms, high incidence of sleep disorders, and anxiety.

Depression is estimated to affect 350 million people. The World Mental Health Survey conducted in 17 countries found that on average about 1 in 20 people reported having an episode of depression in the previous year. Depression is a leading cause of disability worldwide (in terms of total years lost due to disability), especially in high-income countries, ranging from 3% in Japan to 17% in the US (Table 1) [10]. In most countries, the number of people who would suffer from depression during their lives falls within an 8–12% range [11, 12], suggesting significant increased rates of depression in high-prevalence populations (i.e., the US population) than large-sample estimates from the 1980s and 1990s [13]. Furthermore, more recent studies reported that prospectively observed cumulative prevalence of depression resulted nearly twice as high as the lifetime prevalence of major depressive episodes reported by cross-sectional studies during the same time period [14]. Nevertheless, the mental health budgets of the majority of countries constitute less than 1% of their total health expenditures. More than 40% of countries have no mental health policy and over 30% have no mental health programs [15]. Moreover, both direct economic costs of depression in terms of cost of treatment and indirect costs through lost days of work and reduced productivity represent a major issue for public health operators [16].

### 2.2. Depression and Diet, the Association with Fish Consumption

Mental, physical, and social health represents fundamental components for the general well-being of a person. These factors are closely interwoven and deeply interdependent. For instance, the increased prevalence of depression over last decades in Western countries has been accompanied by parallel increased prevalence of cardiovascular diseases and fundamental changes in dietary habits [16, 17]. Several studies suggest that depression may share common pathophysiologic characteristics with cardiovascular diseases and their risk factors [18], such as the increased production of proinflammatory cytokines [19], endothelial dysfunction [20], and elevations in plasma homocysteine levels [21]. Depressive and cardiovascular disorders share blood flow abnormalities (i.e., in depression, hypoperfusion in the limbic system and prefrontal cortex) [22] and decreased glucose metabolism (i.e., low glucose utilization in a number of brain regions correlating negatively with severity of depression) [23]. Given the increases in prevalence of both depression and cardiovascular diseases, it has been hypothesized that a common underlying environmental influence may account for these changes. A comprehensive causal pathway of the relationship between depression and cardiovascular diseases included behavioral and genetic mechanisms [24]. One factor that could explain the relationship between such diseases and explain this parallel increase is the significant shift over the last century in the dietary intake of long-chain PUFA towards an increase in saturated fat and an increase in the ratio of omega-6 to omega-3 fatty acids [25]. Omega-3 PUFA have been reported to both inhibit endothelial cell proliferation [26] and influence glucose uptake [27, 28] and utilization [29] in the brain cells by reducing the expression of both isoforms of the brain glucose transporter GLUT1 in rats [30].

The fatty acid composition of the modern Western diet has changed dramatically during the last century, being characterized by an excessive amount of omega-6 PUFA and a very high omega-6/omega-3 ratio. This pattern of fatty acids intake is thought to promote the pathogenesis of many inflammatory-related diseases, including cardiovascular disease, cancer, and autoimmune diseases, whereas increased levels of omega-3 PUFA and a low omega-6/omega-3 ratio may exert suppressive effects [31]. The increased intake of saturated fatty acids and n-6 essential fatty acids and the reduced consumption of foods containing omega-3 fatty acid, which may exert anti-inflammatory properties, have been hypothesized to correlate with depressive and cardiovascular diseases, increasing the incidence of both disorders [32].

The main sources of fatty acids may vary greatly among countries, mostly depending on food availability and cultural influences. Per capita consumption of EPA and DHA in the US has been reported to be about 50 mg and 80 mg/day, respectively [33]. Evidence from prospective secondary prevention studies suggests that EPA/DHA supplementation ranging from 50 to 180 mg/day (either as fatty fish or supplements) significantly reduces subsequent cardiac and all-cause mortality. For ALA, total intakes of 150 to 300 mg/day seem to be beneficial. Dietary Guidelines suggest including at least two servings of fish per week (particularly fatty fish). In addition, the data support inclusion of vegetable oils (i.e., soybean, canola, walnut, and flaxseed) and food sources (i.e., walnuts and flaxseeds) high in ALA in a healthy diet for the general population [34]. A joint expert consultation of the United Nations Food and Agriculture Organization (FAO) and the World Health Organization (WHO) recommends an intake of 1-2 servings of fish, where each serving is defined as providing 200 to 500 mg/week DHA and EPA [35]. Further recommendations by the National Health and Medical Research Council (NHMRC) issued Nutrient Reference Values for Australia and New Zealand Including Recommended Dietary Intakes, which recommended an intake of combined DHA, EPA, and DPA of 610 mg/day for men and 430 mg/day for women to prevent chronic disease [36].

**Figure 2:**
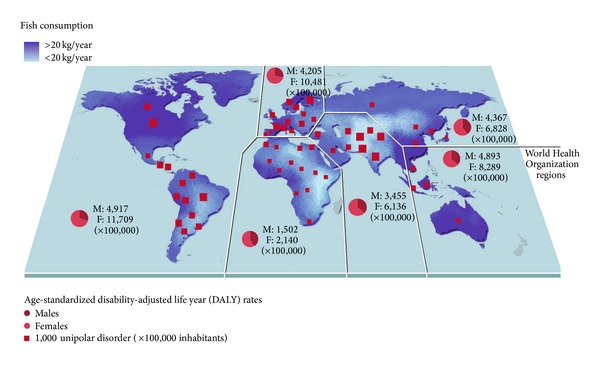
Per capita annual fish consumption and age-standardized disability-adjusted life year for unipolar disorder distribution across countries. DALY rates by gender are also reported per all World Health Organization regions (year 2004). High-income regions reported higher rates of DALY despite their increased consumption of fish, suggesting the role of social environment in the establishment of unipolar depressive disorder. Source: Consumption of Fish and Fishery Products, Fishery and Aquaculture Department 2011, Food and Agriculture Organization of the United Nations (FAOSTAT); the Global Burden of Disease: 2004 Update, World Health Organization, Geneva, 2008.

**Figure 3:**
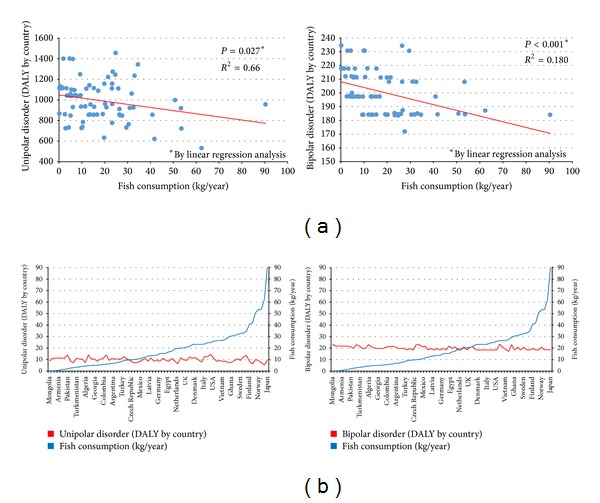
(a) Association between per capita annual fish consumption and age-standardized disability-adjusted life year for unipolar and bipolar disorders by country (year 2004). (b) Countries ordered by increasing fish consumption and relative depressive disorders trends. Both types of graphs demonstrate that DALY rates for unipolar and bipolar disorders are decreased in countries with increased fish consumption. Source: Consumption of Fish and Fishery Products, Fishery and Aquaculture Department 2011, Food and Agriculture Organization of the United Nations (FAOSTAT); the Global Burden of Disease: 2004 Update, World Health Organization, Geneva, 2008.

**Figure 4:**
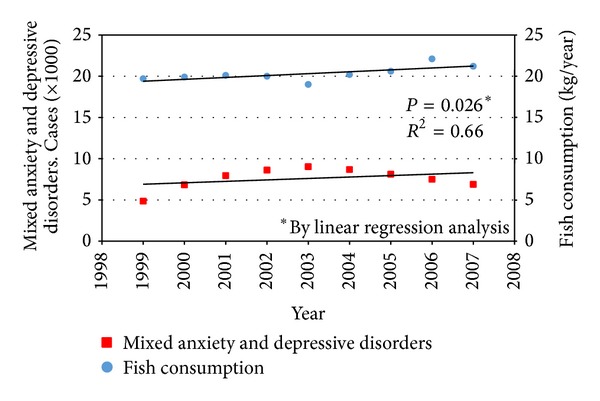
Trend over time (1999–2007) of per capita annual fish consumption and mixed anxiety and depressive disorders in the United Kingdom. Despite a diametric-shaped distribution of cases and relative fish consumption, a significant increasing trend has been found. Source: Consumption of Fish and Fishery Products, Fishery and Aquaculture Department 2011, Food and Agriculture Organization of the United Nations (FAOSTAT); to Walters et al. [3].

The first observation of an inverse association between per capita fish consumption and national annual prevalence of major depression across nine countries was reported about 15 years ago [37]. Since then, several epidemiological studies on oily fish consumption and depression reported a significant inverse correlation between intake of oily fish and prevalence [38–43] and incidence [44–46] of depression and bipolar disorder [47], setting a threshold of vulnerability of about 650 mg/day. We performed an analysis using the data by the Food and Agriculture Organization of the United Nations (FAOSTAT) [48] regarding the total and marine fish consumption by country and the last report of the WHO regarding the global burden of disease, including unipolar and bipolar depressive disorders [10] (Figure 2). Matching together these datasets, we found an inverse correlation between the fish consumption and the age-standardized disability-adjusted life year (DALY) rates for both unipolar and bipolar depressive disorders (Figure 3). A specific analysis of the same variables from 2000 to 2007 in the United Kingdom [3] resulted in a significant inverse correlation between fish consumption and mixed anxiety and depressive disorders and in a significant trend of increased prevalence over time of such disorders (Figure 4). As observed in Figure 2, despite the high consumption of fish, increased rates of depression and/or depressive symptoms have been reported in Western countries. Besides in industrialized countries, such as US and Japan, where stressful lifestyles and the condition of the society may counteract the potential beneficial effects of high fish consumption and increase the overall morbidity burden of depression, in the most of other countries fish consumption seems to correlate with the DALY. It has been hypothesized that this finding might be related to the low quality of diet consumed, especially in such countries [49–53]. Regarding the Mediterranean countries, several studies reported a decreased prevalence [54] and incidence [55–57] of depression and/or depressive symptoms in subjects more adherent to the whole Mediterranean dietary pattern, which include a higher consumption of fish. The favorable effects of the Mediterranean diet on mental health may depend on the synergic positive actions of a variety of foods with a high content of PUFA, such as oily fish [58]. Such positive effects on mental health of long-chain fatty acids contained in the Mediterranean diet also translate the numerous evidences of the protection of such dietary pattern against cardiovascular diseases [59]. However, these conclusions are still not definitive, since other components of the Mediterranean dietary pattern, such as B vitamins, or other fish nutrients, such as iodine and selenium, may exert considerable positive effects on the brain and have a protective role against depression undermining the forthcoming evidence regarding omega-3 fatty acids [60].

## 3. Evidence of Efficacy of Omega-3 Consumption against Depression

### 3.1. Epidemiological Studies

Data from cross-sectional studies exploring the association between omega-3 dietary intake and the prevalence of depression are of a various and contrasting nature. Several studies reported inconclusive results, especially in nonclinical populations [61, 62]. An inverse relationship between intake of omega-3 intake and depression was observed in some studies, although after adjusting for other lifestyle confounders the relationship was no longer significant, suggesting that the relation between depressed mood and omega-3 fatty acids intake may reflect a wider association between depressed mood and lifestyle [63, 64]. Conversely, other cross-sectional studies reported a significant association between omega-3 fatty acids intake and depressive symptoms [43, 65, 66].

Similar discrepancies occur also in prospective cohort studies. A population-based study conducted on 29,133 men of 50 to 69 years living in Finland reported no associations between the dietary intake of omega-3 fatty acids or fish consumption and depressed mood, major depressive episodes, or suicide [67]. Other inconclusive results regarding the association between omega-3 fatty acids and depression were reported in two minor population-based surveys (<1000 subjects) conducted in Australia [68] and Greece [69], although in the Greek one the 90th percentile of the GDS score (towards the high end) exhibited significant negative associations with monounsaturated fatty acids (MUFA) and olive oil [69]. Data from the nationwide Health 2000 Survey (*n* = 5492) and the Fishermen Study on Finnish professional fishermen and their family members (*n* = 1265) revealed a potential protective effect of general fish intake rather than intake or serum concentrations of omega-3 PUFA, although the associations were strongly influenced by lifestyle factors (i.e., high alcohol intake, occasional smoking, or having intermediate physical activity) [41]. Similarly, in a study conducted on 54,202 Danish women followed for 1 year postpartum, a higher risk of postpartum depression was found for the lowest compared with the highest fish intake group, but no association was observed with respect to omega-3 PUFA intake [70]. Despite such contrasting results, high levels of depressive symptoms during pregnancy were reported in women with low omega-3 fatty acid intake from fish [71] and high omega-6/omega-3 ratio [72], especially when results were adjusted for lifestyle factors (i.e., current smokers and women of single marital status) [61]. Results of a large longitudinal study conducted on 54,632 US women from the Nurses' Health Study who were 50–77 years of age and free from depressive symptoms at baseline did not support a protective effect of long-chain n-3 from fish on depression risk after 10 years of followup but support the hypothesis that higher ALA and lower linoleic acid intakes reduce depression risk [73]. On the contrary, results of a study conducted on a subsample from the French Supplementation with Antioxidant Vitamins and Minerals (SU.VI.MAX) cohort followed for 2 years showed that subjects consuming fatty fish or with an intake of long-chain omega-3 PUFA higher than 0.10% of energy intake had a significantly lesser risk of any depressive episode and of recurrent depressive episodes [46]. A recent update from a cohort retrieved by the same study reported no association between omega-3 PUFA intake and incidence of depressive symptoms; an association was observed in cross-sectional analyses, which may reflect unhealthy dietary patterns among subjects with depressive symptoms [74]. Also a study performed in 7,903 participants to the SUN cohort study followed for 2 years suggested a potential benefit of omega-3 fatty acids intake on mental disorders, although no linear trend was apparent [45].

Some studies also considered suicide as a proxy of severe depression and the relationship of suicide rates to omega-3 PUFA and fish consumption. It has been observed that attempters [75] and suicide [76] ate significantly less fish and lower intake of overall PUFA [77], but other studies did not support a protective role of higher intake of fish, EPA, or DHA against suicide [78]. The uncertainty of the results obtained from both cross-sectional and prospective studies on omega-3 fatty acids consumption and prevalence and incidence of depression may depend on the limitations of the methodology used. Indeed, cross-sectional studies do not allow demonstrating a causal relationship between the factors studied because the temporal variable is lacking. On the other hand, prospective studies may suffer by misclassification of exposure, since omega-3 dietary intake was assumed to be constant over the entire follow-up periods. Finally, omega-3 estimation methods by food frequency questionnaires may lead to recall biases in both types of studies.

### 3.2. Experimental Studies

Although current evidence increasingly supports an inverse association between omega-3 PUFA and depression, the validity of findings from experimental research is limited by several methodological issues. Previous meta-analytic studies reported a general positive effect of omega-3 PUFA intake on ameliorating symptoms of depression [79, 80]. On the other hand, incongruent results have been reported in other systematic revisions of the literature [81] and in an updated analysis [82]. The reasons for such variability in these findings depend on the significant heterogeneity among studies examined, weakening the results of the analyses. It has been pointed out that publication bias, unstandardized depression assessment, variability of omega-3 regime employed, and duration of the trial may have affected the analysis. The main limitation of the pooled analysis relied on the selection of studies to be included, taking into account that pathophysiological processes of depressive symptoms involved in MDD patients are likely to be very different from those in patients with depression occurring in other clinical conditions (i.e., bipolar disorder, pregnancy, primary diseases other than depression) and in nonhomogenous patients (i.e., community sample of individuals). The substantial inefficacy of omega-3 PUFA in patients with bipolar disorder or perinatal depression may depend on the fact that the specific pathophysiological processes occurring in MDD patients (which omega-3 PUFA are supposed to affect) are lacking. To the same extent, comorbid depression secondary to CVD, Alzheimer, and schizophrenia may strongly depend on the primary disease. Indeed, besides the differences in omega-3 PUFA regime, length of the trial, and overall quality of the study, it seems that type of diagnosis (i.e., MDD versus depressed mood without diagnosis by DSM-IV criteria) and the homogeneity of the population study (i.e., MDD diagnosed patients versus volunteers recruited by shopping malls, radio and television advertising, and newspapers) were the characteristics mostly affecting the efficacy of the treatment with omega-3 PUFA in these pooled analyses [83].

Some meta-analyses focused on the type of fatty acid used, resulting in a positive effect on depressive symptoms of EPA rather than DHA content of the regime [84, 85]. The most recent meta-analysis of clinical trials concluded that supplements containing EPA ≥60% of total EPA + DHA, in a dose range of 200 to 2,200 mg/d of EPA in excess of DHA, were effective against primary depression [86]. It has been also reported that the more severe was the depression, the more likely omega-3 PUFA supplementation would reduce depressive symptoms.

## 4. Hypothesized Mechanisms of Action

Although epidemiological data and clinical trials suggest that omega-3 PUFA may have preventive and therapeutic effects on depression, the underlying mechanisms are still unclear. The protective role of omega-3 fatty acids against depression has been hypothesized to depend on the physiological mechanisms in which fatty acids take part.

### 4.1. Neuroendocrine Modulation of Omega-3 PUFA in Depression

The pathophysiology of depression has been dominated by the monoamine hypothesis, suggesting that an imbalance, mainly in serotonergic and noradrenergic neurotransmission, is at the core of the pathophysiology of depression. The current therapeutic strategies against depression include drugs which enhance either serotonergic neurotransmission (i.e., selective serotonin reuptake inhibitors (SSRI)), noradrenergic neurotransmission (i.e., noradrenergic reuptake inhibitors (NARI)), or both (i.e., tricyclic antidepressants and more recently serotonin noradrenaline reuptake inhibitors (SNRI)) [87]. However, in 30% of the cases, there is little or no response to the medication, and almost half of patients treated with current antidepressant drugs do not show significant clinical improvements [87].

An effect of omega-3 intake suggested to positively influence the depressive status is the potential interaction with the serotoninergic and dopaminergic transmission, including metabolism, release, uptake, and receptor function. The highly unsaturated nature of EPA and DHA provides them with the quality of highly influencing membrane order (namely the fluidity) of several types of cells [88]. Omega-3 PUFA also regulate the signal transduction by enhancing G-protein-mediated signal transduction [89, 90], membrane-bound enzymes (Na/K-dependent ATP'ase) [91], and protein kinase C [92]. The membrane changing induced by omega-3 PUFA intake may affect different neurotransmitter system altering the regulation of dopaminergic and serotonergic neurotransmission, which are dysfunctional in depressed patients. Changes in serotonin (5-HT) and dopamine receptor (DR-2) number and function caused by changes in PUFA provide the theoretical rationale connecting fatty acids with the current receptor and neurotransmitter theories of depression. Cerebrospinal fluid (CSF) 5-hydroxyindoleacetic acid (5-HIAA), a metabolite that reflects serotonin turnover, has been reported to be decreased in several psychiatric conditions, including violent suicide attempts during depression [93]. It has been reported that higher concentrations of plasma DHA predict an increase in serotonergic neurotransmission (higher CSF 5-HIAA) in healthy adults [94] and in an experimental animal model of depression [95]. Conversely, omega-3 deficiency results in an increase of serotonin receptor (5HT2) density in the frontal cortex, probably due to an adaptation to reduced serotoninergic function [96, 97]. A preclinical animal experiment reported that erythrocyte DHA was inversely correlated, and AA and the AA/DHA and AA/EPA ratios were positively correlated with plasma IL-6, TNF*α*, and CRP levels, whereas plasma IL-6 levels were positively correlated with 5-HIAA/5-HT ratios in all brain regions, providing evidence for a functional link between n-3 fatty acid deficiency, elevated peripheral inflammatory signaling, and increased central 5-HT turnover [98].

Regarding the dopamine neurotransmission, in animal experimental models of depression, decreased levels of dopamine turnover in the prefrontal cortex and dopamine levels up to 6-fold higher in the nucleus accumbens have been reported [99, 100]. Similar observations were reported in omega-3 PUFA-deficient rats, in which the expression of the dopamine receptor (D2R) was decreased in the frontal cortex and increased in the nucleus accumbens [96, 101, 102]. Conversely, supplementing the diet of rats with omega 3 PUFA led to a 40% increase in dopamine levels in the frontal cortex as well as an increase in the binding to the dopamine D2 receptor [103].

Beside the well-known deficiency in serotonergic neurotransmission as pathophysiological correlate of major depression, recent evidence points out to an important role of increased glutamate receptor activation as well [104]. Indeed, an increased activity of the glutamatergic system and N-methyl-D-aspartate (NMDA) receptor agonism has been associated with depressed mood, whereas a reduction of the glutamatergic activity may exert antidepressant action. These effects of the glutamatergic system on mood may depend on its direct or indirect influence on the serotonergic and noradrenergic neurotransmission, since NMDA receptor antagonists increase the serotonin levels in the brain [105, 106]. Omega-3 deficiency has been demonstrated to promote age-induced degradation of glutamatergic transmission and its associated astroglial regulation in the hippocampus [107] by slowing astroglial glutamate transport via a specific signal-like effect [108]. Further experimental models confirmed that dietary omega-3 content is relevant for the glutamatergic system development and for behavioral performance in adulthood [109]. At a molecular level, it has been demonstrated that the NMDA receptor can be stimulated by the protein kinase C, whose conformational changes and optimal activation depend on for membrane content of omega-PUFA [110, 111]. The putative correlation among the neurochemical and behavioral alterations caused by dietary omega-3 PUFA and glutamatergic transmission must be further investigated in future research.

Glucocorticoids play a fundamental role in attenuating the inflammation processes following exposure to a variety of stress-related conditions [112]. Glucocorticoids suppress critical inflammatory signaling pathways including nuclear factor-*κ*B (NF-*κ*B) and inhibit stress-related outflow pathways including the corticotropin releasing hormone (CRH), the hypothalamic-pituitary-adrenal (HPA) axis, and the sympathetic nervous system [113]. Failure of glucocorticoids to inhibit inflammatory and neuroendocrine responses to challenge may contribute to disease development, although the etiology of glucocorticoid resistance in both inflammatory and neuropsychiatric disorders is unknown. Depression has been associated with a high level of cortisol in blood due to the hyperactivity of HPA axis, largely due to a hypersecretion of CRH [104]. EPA may regulate the HPA axis dysfunction associated with depression by reducing corticotrophin releasing factor expression and corticosterone secretion [114]. Some animal studies reported that the response to chronic stress can be modulated by the omega-3 fatty acid supply, since a dietary deficiency has been found to be deleterious while enrichment has protected against stress [115]. These effects were associated with the reduction of corticosterone levels promoted by the PUFA supplementation in the stress-induced animals [116, 117]. From a mechanistic point of view, it has been demonstrated that omega-3 PUFA inhibit the P-glycoprotein (P-gp) activity [117], which are transport proteins responsible of the increase in cortisol transport through the blood-brain barrier (BBB) in depressive subjects [118–122]. The normalization in brain penetration of cortisol would normalize the feedback control of the HPA axis. Another study demonstrated a modulatory effect of omega-3 PUFA by increasing the cortisol transport in the BBB models not through the inhibition of P-gp efflux, but thanks to membrane fluidification and some effect on tight junction integrity [123].

### 4.2. Anti-Inflammatory Effects of Omega-3 PUFA

Recent studies indicate that factors other than monoamine deficiency or hyperactivation of the HPA axis must be considered when examining the pathogenesis of major depression such as an altered activation of immune system and chronic inflammation with a specific impairment in the signaling of neurotrophins, such as transforming growth factor *β*1 (TGF-*β*1) [124, 125].

According to recent evidence, chronic stress can elicit a neuroinflammatory response through the activation of microglia in CNS, with ensuing release of inflammatory mediators such as interleukin-1*β* (IL-1*β*) and tumor necrosis factor-*α* (TNF-*α*) [126]. The neuroinflammatory response leads to inhibition of neurotrophin signaling and can also elicit both sickness behavior and psychological pain. In addition, chronic stress alters activation of immune system in the periphery, which might account for the state of chronic inflammation observed in depressed patients [127]. Different studies have demonstrated a positive correlation between the severity of the symptoms of depression and the increase in the inflammatory status [127]. Proinflammatory cytokines interfere with many of the pathophysiological mechanisms that characterize the pathogenesis of depression, altering serotonin metabolism, and reducing both synaptic plasticity and hippocampal neurogenesis [127]. On the other hand, reduced levels of anti-inflammatory cytokines, such as interleukin-4 (IL-4), interleukin-10 (IL-10), and TGF-*β*1, have been found in the plasma of depressed patients [127, 128].

Chronic systemic inflammation also contributes to the progression of neurodegeneration [129]. The key anti-inflammatory effect of omega-3 fatty acids has been long recognized to depend on their action on eicosanoids. Eicosanoids are biologically active lipid mediators produced from PUFA which play a role in inflammation and regulation of immune function [130]. To produce these eicosanoids, AA is released from membrane phospholipids through the action of phospholipase A2 enzymes and then acts as a substrate for cyclooxygenase (COX), lipoxygenase, or cytochrome P450 enzymes. COX enzymes lead to PG and thromboxanes, lipoxygenase enzymes lead to leukotrienes (LT), and cytochrome P450 enzymes lead to hydroxyeicosatetraenoic and epoxyeicosatrienoic acids. Omega-3 EPA and DHA incorporation in cell membrane decreases their AA content and reduces the amount of substrate available to produce inflammatory and immunoregulatory eicosanoids [131]. LTB5, a product of EPA, is a competitive antagonist to LTB4, a highly proinflammatory eicosanoid derived from AA [132]. A series of studies gave important information regarding the omega-3 fatty acids as mediators of inflammatory response in depressive status. Indeed, it has been demonstrated that severity of depression varies with the degree of omega-3 fatty acids in erythrocyte membranes, which are decreased in more severe status, as an indicator of oxidative damage [133–136]. It has been also reported that plasma fatty acids composition and depression are associated with a significant higher ratio of omega-6 to omega-3 PUFA in depressed subjects [137–140]. Many studies also focused on analysis of fasting bloods for detection of plasma fatty acid analysis in risk population. Results from a case-control study conducted on 16 depressed and 22 nondepressed women recruited during the third trimester of pregnancy demonstrated that high DHA, high total n-3, and a low n-6 : n-3 ratio were associated with significantly lower odds of depression [141]. Similar findings were reported in some studies conducted on depressed postmyocardial infarction [142] and acute coronary syndromes patients [143, 144] in which, compared with control group, lower levels of long-chain omega-3 PUFA as measured by a mean AA/EPA ratio were found. Moreover, a low DEA percentage and low omega-3 proportions of lipid profile predicted risk of suicidal behavior among depressed patients over the 2-year period [145]. Other evidences come from a case-control study conducted on 150 subjects reporting an association between fatty acids with serotonergic and immunological markers in depressive patients but not in patients with somatization syndrome suggesting a different biological mechanism of depression and somatoform disorders [146]. This may lead to the speculation of a potential bias in previous studies on depression assessment concerning the indiscriminate merging together of both disorders that could affect the outcome. Similarly, an association between omega-3 fatty acids in adipose tissue and major depression has been shown [147–149], although not univocally reported [150, 151].

Dysregulation of the functional activity of the immune system in depression is a phenomenon that has been widely reviewed [152]. As discussed above, the peripheral immune activation observed in major depression, through the release of proinflammatory cytokines, is responsible for the variety of behavioral, neuroendocrine, and neurochemical alterations that are associated with this psychiatric condition [152]. Depression has been associated with excessive production (during an acute phase response) of proinflammatory cytokines, such as IL-1 beta, IL-12, and interferon-gamma. A recent meta-analysis of experimental studies reported a significantly higher concentration of the proinflammatory cytokines tumor necrosis factor-alpha and IL-6 in depressed subjects compared with control subjects [153]. The actions of omega-3 on cells include the changing of the expression of key cell surface proteins and the modulation of the production of proinflammatory cytokines. Indeed, omega-3 PUFA have been reported to decrease production of TNF, IL-1b, and IL-6 in* in vitro* studies and decrease production of TNF, IL-1b, IL-6, and various growth factors in healthy human subjects, although not all studies confirm this effect [154]. At the cellular level, they have been demonstrated to decrease activation of NF-*κ*B, a key transcription factor involved in upregulation of inflammatory cytokine [154]. The question arises as to whether the decreased prevalence of depressive symptoms accompanying the higher plasma content of omega-3 PUFA is also associated with improved central inflammation, that is, cytokine activation, in the brain. Recent studies have pointed out the possible role of omega-3 PUFA inducing a central antidepressant-like effect by modulating oxidative reactions and inflammatory cytokine production in microglial and neuronal cells. This determines a reduction of expressions of tumor necrosis factor-*α*, interleukin-6, nitric oxide synthase, and cyclooxygenase-2, an induction by interferon-*γ*, and an induction of upregulation of heme oxygenase-1 (HO-1) in BV-2 microglia [155]. However, results of experimental studies on cytokines response after administration of omega-3 fatty acids are not univocal. For example, long-term intake of omega-3 increased plasma serotonin concentration and the hippocampus c-AMP response element binding protein (CREB) and reducing interleukin-6 (IL-6) expression in rats, but clear dose-dependent effects and significant differences in expressions of IL-1*β*, tumor necrosis factor-*α*, brain-derived neurotrophic factors, or phosphorylated CREB were not found [156]. Moreover, another experimental study on mice demonstrated that high level of brain DHA was associated with a decrease in depressive-like symptoms throughout aging independently on the cytokines response (in fact, increased interleukin-6 and decreased IL-10 expressions were found in the cortex of aged mice independently of the diets) [157].

Among the anti-inflammatory actions of omega-3, it is noteworthy that they have been recently discovered as a source of docosanoids, metabolites with a novel stereospecificity unlike that of the known eicosanoids [158]. The three known classes, namely, docosatrienes, resolvins, and protectins, are produced mainly from controlled oxidative breakdown of DHA within the membrane and demonstrated anti-inflammatory properties [159]. Novel research on depression focused on the role of resolvins, which are thought to terminate ongoing inflammatory cascades and may be responsible for the potential anti-inflammatory effects of omega-3 PUFA in preventing or ameliorating the depressive status [160]. Resolvins are grouped into E-series and D-series, depending on if derived by EPA or DHA, respectively. Resolvin E1 has been reported to reduce inflammation by suppressing the activation of the transcription factor nuclear factor-*κ*B and subsequent synthesis of inflammatory cytokines and chemokines [160].

As discussed above, major depression is characterized by increased levels of proinflammatory cytokines and reduced levels of anti-inflammatory cytokines such as IL-10 and TGF-*β*1 [124, 127]. Plasma TGF-*β*1 levels are reduced in major depressed patients and show a significant negative correlation with the Hamilton Depression Rating Scale [128, 161, 162]. Interestingly, TGF-*β*1 levels significantly increase after antidepressant treatment [128], and SSRI drugs such as sertraline might exert immunomodulatory effects* in vivo* through a decrease in the proinflammatory cytokine IL-12 and an increase in the anti-inflammatory cytokines such as IL-4 and TGF-*β*1 [162]. Similarly, therapeutic concentrations of venlafaxine prevent microglial activation, reduce proinflammatory cytokine secretion, and finally increase the release of TGF-*β*1 in an astroglia-microglia coculture model [163]. Recent studies suggest that omega-3 fatty acids can increase both* in vitro *and* in vivo* the synthesis of TGF-*β*1 [164, 165] and, in particular, in pregnant women [166], although no studies have been yet conducted in depressed patients. On the basis of this evidence, it might be worth assessing whether TGF-*β*1 signaling is a common target both for omega-3 fatty acids and antidepressant drugs, and whether omega-3 fatty acids can exert their antidepressant* in vivo* effects via the rescue of TGF-*β*1 signaling.

## 5. Conclusions

The role of omega-3 in preventing psychiatric diseases remains to be clarified. It can be speculated that all types of action can occur simultaneously: on one hand, by maintaining and increasing the brain structures and preserving their function by interacting with phospholipid metabolism and, hence, the modulation of signal transduction; on the other hand, preventing or decreasing the inflammatory status occurring during depression. However, the problem of how to correct the inadequate supply of omega-3 fatty acids in Westernized countries' diet is a priority in order to set food and health policies and dietary recommendations for individuals and population groups. Moreover, accompanying the increased dietary intake of omega-3 fatty acids, an omega-6/omega-3 ratio maintained not above 5 is highly desirable. If omega-3 PUFA will result to be effective for both the prevention and treatment of depression, substantial implications with large-scale impact through dietary interventions could be realized. Although many other factors may also contribute to the rise in depression and for which effective (although not efficient) treatments already exist, dietary recommendations suggesting proper intake of omega-3 PUFA and dietary interventions including omega-3 PUFA supplement can result in substantial benefits for the general population.

## Figures and Tables

**Table 1:** Age-standardized disability-adjusted life year (DALY) rates by income (*Global Burden of Disease: 2004 Update*, WHO, Geneva, 2008).

	World			Low-income countries			Middle-income countries			High-income countries		
	Disease or injury	DALYs (millions)	% of total DALYs	Disease or injury	DALYs (millions)	% of total DALYs	Disease or injury	DALYs (millions)	% of total DALYs	Disease or injury	DALYs (millions)	% of total DALYs
1	Lower respiratory infections	94.5	6.2	Lower respiratory infections	76.9	9.3	***Unipolar depressive disorders***	29.0	5.1	***Unipolar depressive disorders***	10.0	8.2
2	Diarrhoeal diseases	72.8	4.8	Diarrhoeal diseases	59.2	7.2	Ischaemic heart disease	28.9	5.0	Ischaemic heart disease	7.7	6.3
3	***Unipolar depressive disorders***	65.5	4.3	HIV/AIDS	42.9	5.2	Cerebrovascular disease	27.5	4.8	Cerebrovascular disease	4.8	3.9
4	Ischaemic heart disease	62.6	4.1	Malaria	32.8	4.0	Road traffic accidents	21.4	3.7	Alzheimer and other dementias	4.4	3.6
5	HIV/AIDS	58.5	3.8	Prematurity and low birth weight	32.1	3.9	Lower respiratory infections	16.3	2.8	Alcohol use disorders	4.2	3.4
6	Cerebrovascular disease	46.6	3.1	Neonatal infections	31.4	3.8	COPD	16.1	2.8	Hearing loss, adult onset	4.2	3.4
7	Prematurity and low birth weight	44.3	2.9	Birth asphyxia and birth trauma	29.8	3.6	HIV/AIDS	15.0	2.6	COPD	3.7	3.0
8	Birth asphyxia and birth trauma	41.7	2.7	***Unipolar depressive disorders***	26.5	3.2	Alcohol use disorders	14.9	2.6	Diabetes mellitus	3.6	3.0
9	Road traffic accidents	41.2	2.7	Ischaemic heart disease	26	3.1	Refractive errors	13.7	2.4	Trachea, bronchus, lung cancers	3.6	3.0
10	Neonatal infections and other^b^	40.4	2.7	Tuberculosis	22.4	2.7	Diarrhoeal diseases	13.1	2.3	Road traffic accidents	3.1	2.6

COPD: chronic obstructive pulmonary disease.
